# Advanced Imaging Techniques for Atherosclerosis and Cardiovascular Calcification in Animal Models

**DOI:** 10.3390/jcdd11120410

**Published:** 2024-12-22

**Authors:** Lifang Ye, Chih-Chiang Chang, Qian Li, Yin Tintut, Jeffrey J. Hsu

**Affiliations:** 1 Heart Center, Department of Cardiovascular Medicine, Zhejiang Provincial People’s Hospital (Affiliated People’s Hospital, Hangzhou Medical College), Hangzhou 310014, China; lifangye@mednet.ucla.edu; 2 Department of Medicine, University of California, 650 Charles E Young Dr. S, Center for Health Sciences, Room A2-237, Los Angeles, CA 90095, USA; changc4@ucla.edu (C.-C.C.); qiali@mednet.ucla.edu (Q.L.); ytintut@mednet.ucla.edu (Y.T.); 3 Department of Bioengineering, University of California, Los Angeles, CA 90095, USA; 4 Department of Physiology, University of California, Los Angeles, CA 90095, USA; 5 Department of Orthopedic Surgery, University of California, Los Angeles, CA 90404, USA; 6 Department of Medicine, Veterans Affairs Greater Los Angeles Health Care System, Los Angeles, CA 90073, USA

**Keywords:** cardiovascular calcification, computed tomography (CT), positron emission tomography (PET), magnetic resonance imaging (MRI), light-sheet fluorescence microscopy, photoacoustic imaging, animal models, molecular imaging

## Abstract

The detection and assessment of atherosclerosis and cardiovascular calcification can inform risk stratification and therapies to reduce cardiovascular morbidity and mortality. In this review, we provide an overview of current and emerging imaging techniques for assessing atherosclerosis and cardiovascular calcification in animal models. Traditional imaging modalities, such as computed tomography (CT) and magnetic resonance imaging (MRI), offer non-invasive approaches of visualizing atherosclerotic calcification in vivo; integration of these techniques with positron emission tomography (PET) imaging adds molecular imaging capabilities, such as detection of metabolically active microcalcifications with ^18^F-sodium fluoride. Photoacoustic imaging provides high contrast that enables in vivo evaluation of plaque composition, yet this method is limited by optical penetration depth. Light-sheet fluorescence microscopy provides high-resolution, three-dimensional imaging of cardiovascular structures and has been used for ex vivo assessment of atherosclerotic calcification, but its limited tissue penetration and requisite complex sample preparation preclude its use in vivo to evaluate cardiac tissue. Overall, with these evolving imaging tools, our understanding of cardiovascular calcification development in animal models is improving, and the combination of traditional imaging techniques with emerging molecular imaging modalities will enhance our ability to investigate therapeutic strategies for atherosclerotic calcification.

## 1. Introduction

Cardiovascular calcification is used as a surrogate marker of atherosclerosis, and while previously considered a passive event, a variety of active mechanisms have now been implicated in its development in plaque and the artery wall [1]. This process leads to arterial stiffening and impacts atherosclerotic plaque vulnerability, which is associated with the morbid and potentially fatal cardiovascular events of myocardial infarction (MI) and stroke [2,3]. The presence of calcification within atherosclerotic plaque, particularly “spotty” clusters of small calcium deposits, increases the risk of plaque rupture in part due to mismatch of tissue compliance at the interface between the calcified deposits and surrounding tissue [4]. The ability to identify these vulnerable plaques facilitates the detection of individuals at increased risk of cardiovascular events caused by plaque rupture [5,6,7].

Advancements in vascular plaque imaging have improved the ability to non-invasively detect and characterize atherosclerotic plaque. Traditional imaging methods, such as computed tomography (CT) and magnetic resonance imaging (MRI), have provided means to assess plaque presence and composition, with CT imaging already widely used clinically for the assessment of coronary artery calcification. However, the resolution of these imaging modalities makes it challenging to detect early microcalcific changes, particularly in animal models [8,9,10]. Advanced imaging techniques, such as positron emission tomography (PET) and the use of novel molecular tracers, are promising with regard to the detection and quantification of atherosclerotic calcification at earlier stages [8,9,10]. These techniques offer additional characterization of plaque features and their evolution over time, as well as allowing for in vivo evaluation of the efficacy of candidate therapies [11,12].

Animal models of cardiovascular calcification allow for mechanistic studies of potential therapeutic interventions. A number of mouse models for atherosclerotic calcification have been used to study this process [13], with a major limitation being the lack of atherosclerotic plaque rupture seen in the vast majority of commonly used models (though there have been recent developments toward models that do feature plaque rupture) [14]. Nonetheless, histological assessment of these animal models has been instrumental in providing insight into atherosclerotic disease development but is limited by the need to sacrifice the animal to study features such as calcification morphology. Imaging modalities of these animal models offer the opportunity to evaluate these features in vivo and assess the response to therapies at the individual level.

The primary goal of this review is to address imaging advancements in the assessment of vascular plaque in preclinical animal models and to provide a summary of the latest imaging methods used. We describe the technological progress that has increased the sensitivity, specificity, and practical utility of these imaging modalities in animals. We apply a narrow framework for the application of these methods to early atherosclerotic disease detection, disease process maturation, and monitoring of the effectiveness of preventive therapies. Further, we highlight the role of these techniques in improving our understanding of atherosclerosis and cardiovascular calcification, including their advantages and disadvantages (Table 1).

## 2. Computed Tomography (CT)

**Figure 1:**
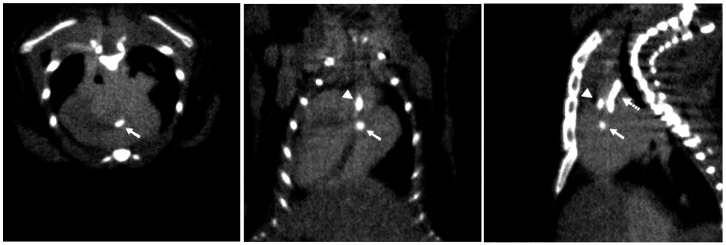
Example of micro-computed tomography (μCT) images showing aortic calcification with the use of an intravenous contrast agent, Omnipaque 350. Transverse (**left**), coronal (**middle**), and sagittal (**right**) cardiac sections display calcium deposits in the regions of the aortic valve (closed arrow), aortic root (arrowhead), and aortic arch (dashed arrow). Adapted with permission from Hsu et al. (Ref. [28]).

Micro-computed tomography (µCT) has emerged as an effective imaging modality in preclinical research on atherosclerosis, providing high resolution and precision in the detection and quantification of cardiovascular calcification [5,6,7,27]. The µCT scanner uses microfocus X-ray sources in conjunction with high-resolution detectors to generate 3D images for volumetric and densitometric assessment of calcium deposits, providing an advantage over the traditional, ex vivo histological methodologies that produce two-dimensional cross-sectional views [6]. While some µCT scanners allow for a spatial resolution as small as 0.4 µm in ex vivo imaging, in vivo µCT systems can detect calcium deposits as small as ~2.4–10 µm (depending on the scanner) within the vasculature [6,7]. Microcalcifications that are less than 50 µm in size are found in the early stages of atherosclerosis [15], whereas macrocalcification, defined as larger deposits greater than 50 µm, are seen in more advanced atherosclerotic disease. Our group has previously used µCT to monitor the progression of aortic valve and aortic calcification in the hyperlipidemic *Apoe^−/−^* mouse model in vivo (Figure 1) [28,64].

In addition to in vivo imaging of calcification, ex vivo µCT imaging of aortic tissues using contrast dyes also provides characterization of atherosclerotic plaque that includes quantification of plaque volume, intimal surface area, and maximum lesion thickness [16,29,31]. Incorporating intravascular contrast agents, such as iodine or phosphotungstic acid (PTA), has augmented these atherosclerotic plaque quantification capabilities of µCT, which has been validated against histological analyses in mice [17,29,31] and in rabbits [18,19]. In the swine model of coronary atherosclerosis, a semiautomatic segmentation technique based on 2D histograms was developed, allowing for the comprehensive visualization and quantification of contrast-agent-free, 3D, high-resolution reconstructions of full-length artery walls [20]. However, while ex vivo scanning helps to enhance resolution, it restricts the ability to conduct longitudinal studies on the same animal subjects [6,7,27].

CT has inherent limitations. A primary concern is the high radiation dose resulting from prolonged scanning, which can potentially compromise the viability and physiology of small animal models [5,6,7]. Furthermore, motion artifacts, arising from cardiac contractions, respiration, and general motility, can lead to image blurring and reduced quality, complicating the accurate assessment of vascular structures [7,17]. The use of contrast agents, while enhancing the image quality, also introduces variability and potential artifacts that can complicate data interpretation [16]. Moreover, µCT does not effectively visualize the internal structure of calcifications, hindering the imaging of intricate features such as calcified cell recesses and cell cracks [5,32].

## 3. Positron Emission Tomography (PET)

**Figure 2:**
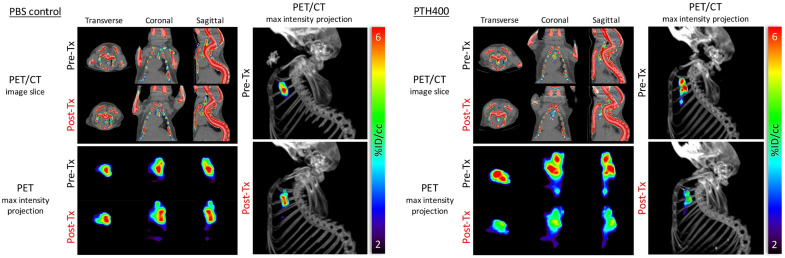
Micro-computed tomography (μCT) and micro-positron emission tomography (μPET) imaging of aortic calcification in mice. This figure presents a comparison between 12-month-old PBS-treated control mice and 16-month-old mice treated with parathyroid hormone (PTH), emphasizing fluoride uptake as an indication of calcium mineral surface area. The top-left panel shows transverse, coronal, and sagittal μCT and μPET slices of the chest, while the bottom-left panel displays maximum-intensity projections (MIPs) of the mediastinal regions of interest from corresponding views. The right panel features a lateral view of the μPET MIP superimposed onto the μCT image, delineating the skeletal structure. Adapted with permission from Hsu et al. (Ref. [28]).

PET imaging, another valuable tool in preclinical cardiovascular research, is a non-invasive imaging technique to assess specific biological processes in animal models, including cardiovascular calcification and atherosclerosis, at the molecular level using radiolabeled tracers designed to target the processes of interest [21,37,65,66,67]. For cardiovascular calcification, the radiotracer ^18^F-NaF is used. ^18^F-NaF binds to hydroxyapatite crystals in microcalcifications, allowing the early detection of calcification activity before significant macroscopic calcification occurs, and our group has previously used ^18^F-NaF micro-PET imaging to assess cardiovascular calcification morphology in mice (Figure 2) [65,68,69]. ^18^F-NaF, which is not taken up by myocardial cells, eliminates obscuring signals from the myocardium and allows the more specific detection of calcification [65]. As for atherosclerotic plaque development and progression [37,65,66], the radiotracer ^18^F-FDG is used. This glucose analog is taken up by the inflammatory regions of atherosclerotic plaque, primarily due to the increased metabolic activity of inflammatory cells by the glucose transporters, mainly GLUT1 [21,40,65,70,71]. Thus, ^18^F-FDG imaging highlights the metabolically active inflammatory cells within the plaque [40,71].

Recent advancements in PET imaging have pushed the boundaries of the detection and assessment of atherosclerotic plaque. An ^18^F-anti-VCAM-1 tracer identified atherosclerotic lesions both in vivo and ex vivo, improving the signal-to-noise ratio using a sub-millimeter PET system [33]. Novel ^18^F-YLF-DW(oxazolo[4,5-b] pyridines and fluorenone compounds) and ^18^F-ASEM(3-(1,4-diazabicyclo[3.2.2]nonan-4-yl)-6-18F-fluorodibenzo[b,d]thiophene 5,5-dioxide) are used to pinpoint inflammation and potential instability in plaque [22,23]. YLF-DW and ASEM are α7-nAChR-selective ligands that target the α7-nAChR, which is expressed in various cells, including neurons and inflammatory cells like macrophages [22,23,24]. In addition, the introduction of a new fibrin-binding PET probe (68Ga-CM246) in an atherosclerotic rabbit model offers a promising approach for imaging high-risk atherothrombosis [41].

The PET imaging in preclinical models has some limitations for translational purposes. The spatial resolution of PET may not fully capture the complexity of human atherosclerotic plaque [21,65]. The development and validation of new radiotracers require extensive time and resources [34,38]. There are also concerns regarding the potential toxicity and safety of novel radiotracers, which must be thoroughly evaluated before clinical application [23,66]. Nevertheless, the continued development and application of new radiotracers, combined with advancements in imaging technology, has potential to aid in the development of targeted therapies for atherosclerosis and cardiovascular calcification.

## 4. Magnetic Resonance Imaging (MRI)

MRI is another non-invasive imaging modality for atherosclerotic plaque, offering detailed visualization without ionizing radiation, making it safe for repeated use [72,73,74,75]. Its applications include assessing plaque composition, inflammation, and progression with advanced techniques such as DCE-MRI, providing additional functional insights, such as inflammation and neovascularization [73,76,77].

**Figure 3:**
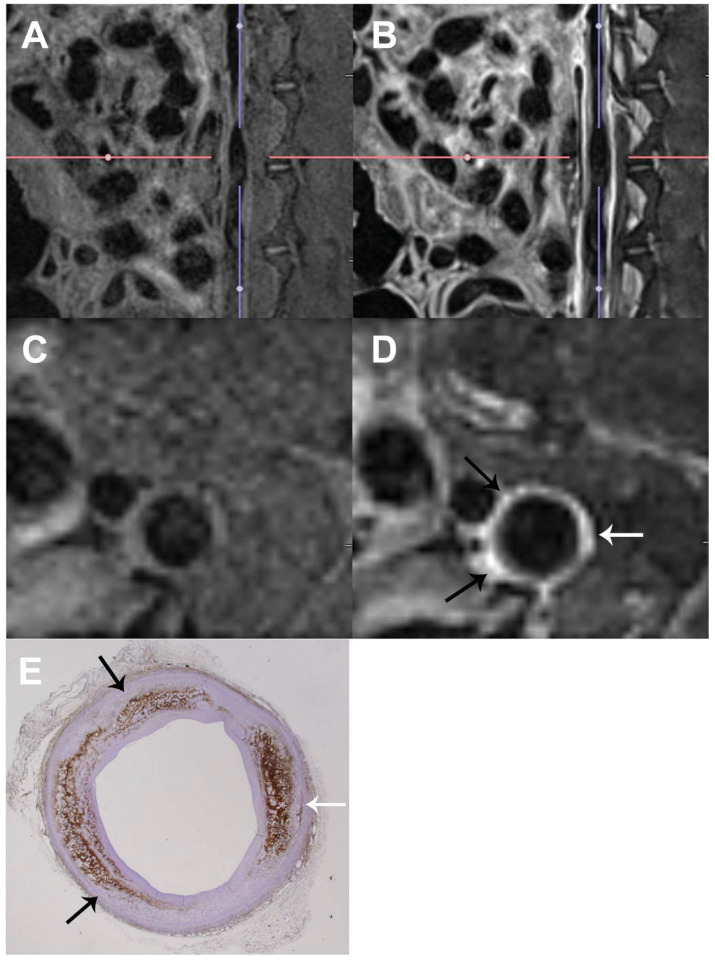
Magnetic resonance imaging (MRI) of aortic atherosclerosis in rabbits. (**A**,**C**) Three-dimensional, high-resolution, pre-T1-weighted MR scan and corresponding transverse slice at the level of the crosshairs. (**B**,**D**) Three-dimensional, high-resolution, post-T1-weighted MRI scan and corresponding transverse slice at the level of the crosshairs. On the post-contrast transverse MRI scan (**D**), the atherosclerotic wall showed marked contrast enhancement (white and black arrows). (**E**) The corresponding histopathologic section (RAM-11-positive staining; magnification ×12.5) demonstrates abundant macrophage accumulation (white and black arrows) in the area matched with the marked contrast-enhanced area on the MR scan. Adapted with permission from Hur et al. (Ref. [80]).

MRI employs strong magnetic fields and radiofrequency waves [72,73], distinguishing between pathological and normal tissues based on their different relaxation properties after being disturbed by a magnetic pulse. In regions of inflammation within atherosclerotic plaque, there is often increased permeability of the vessel wall owing to endothelial dysfunction. This permeability allows the contrast agent to accumulate in the inflamed areas, which can be shown by DCE-MRI as regions with enhanced signal intensity [76,77,78]. The extent and pattern of contrast agent uptake provide information on the degree of inflammation. Plaque showing rapid and significant contrast enhancement is likely to have higher inflammatory activity, which is a marker of plaque instability and vulnerability (Figure 3) [39,46,79,80]. In atherosclerotic plaque, MRI can visualize the vessel wall and the plaque itself owing to differences in water content, blood flow, and tissue composition [47,76]. MRI can help with the characterization of atherosclerotic plaques by detecting their size, composition, fibrous cap, lipid-rich necrotic core, and neovascularization [39,47,76,77,79]. Advanced MRI techniques such as ultrasmall superparamagnetic iron oxide particles target macrophage-rich inflammation within plaque [81].

A key feature of vulnerable atherosclerotic plaque is the development of new blood vessels (neovascularization) within the plaque [82,83]. These neovessels are typically leaky and fragile and contribute to plaque growth and instability. DCE-MRI can detect the presence of neovessels by observing the patterns of contrast enhancement over time. The rate and degree of contrast uptake in plaque are indicative of neovascularization. Plaque with significant neovascularization exhibits early and intense contrast enhancement due to the rapid perfusion of the contrast agent into the newly formed microvasculature [46,47,79]. MR molecular imaging with ανβ3-targeted nanoparticles can serially map angiogenesis in the aortic wall and monitor the progression of atherosclerosis [42,45]. Studies on plaque angiogenesis in atherosclerotic rabbits illustrate the value of DCE-MRI for monitoring disease progression [76,77]. Gadolinium-based contrast agents also enhance visibility, especially for neovascularization [47,76,77,79].

MRI has several limitations. This technology is expensive and not as widely available as other imaging modalities, such as CT or ultrasound [73,75]. The long duration of MRI scans can be uncomfortable for animals, and imaging rapidly moving structures such as the heart can be challenging [72,75]. Although advancements in electrocardiogram (ECG) gating techniques and faster imaging sequences have enabled synchronized cardiac image acquisition, these issues can still affect image quality [72,73,75]. Nonetheless, as detailed below, when combined with other imaging modalities such as PET and CT, MRI can be a helpful tool for the in vivo assessment of atherosclerotic calcification in preclinical animal models.

## 5. Photoacoustic Imaging (PAI)

Photoacoustic imaging is an advanced optical imaging modality and offers several advantages in cardiovascular applications. PAI combines the deep penetration depth of ultrasound imaging with the high contrast and high spectroscopic specificity of imaging [30,35,43]. Its basic principle involves the generation of photoacoustic waves through the absorption of pulsed laser light by biological tissues, followed by the thermoelastic expansion and emission of ultrasonic waves [35,43] that are then used to reconstruct images [30,35,43]. This technique allows a detailed visualization of tissue composition and structure due to specific optical absorption properties of different tissues, making it highly suitable for in vivo applications [36,44,84,85,86,87], such as identifying atherosclerotic plaque and calcification [30,35,43]. For example, lipids, a major component of atherosclerotic plaque, have specific absorption peaks that can be targeted using PAI [30,43]. Additionally, PAI is capable of monitoring blood flow speed, detecting metabolic blood oxygen levels in tissue, and detecting (micro)vasculature networks.

**Figure 4:**
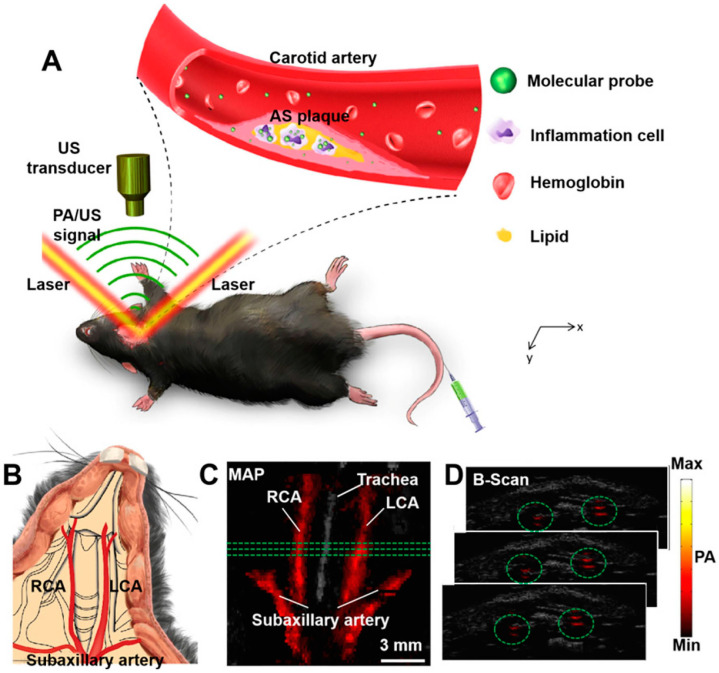
Noninvasive photoacoustic imaging for carotid atherosclerosis detection in mice. (**A**) Illustration of the principle behind detecting carotid atherosclerotic inflammation, where a beam of excitation light targets the neck of a shaved mouse, generating photoacoustic (PA) signals captured by an ultrasonic transducer. (**B**) Schematic anatomy of a mouse’s carotid arteries. (**C**) Fused PA/ultrasound (US) maximum amplitude projection (MAP) image. (**D**) Consecutive B-scan images. The PA signals, highlighted in green circles in (**D**), correspond to the locations of the carotid arteries, marked by three green lines in (**C**). The ultrasound image components are represented in gray, and the photoacoustic image components in hot colors. Ultrasound image: gray; photoacoustic image: hot. AS plaque: atherosclerotic plaque; LCA: left carotid artery; PA: photoacoustic; RCA: right carotid artery; US: ultrasound. Adapted with permission from Xie et al. (Ref. [36]).

Recent studies have collectively underscored the potential of PAI as a powerful tool for the non-invasive longitudinal monitoring of atherosclerotic lesions and inflammation [36,44,84,85,86,87]. In a recent study, semiconducting polymer nanoparticles (PBD-CD36) in combination with PAI were used to noninvasively assess inflammation in carotid atherosclerosis (Figure 4) [36]. This study demonstrated significant photoacoustic signal enhancement in inflamed carotid arteries labeled with the PBD-CD36 probe, correlating with CD36-positive expression areas [36]. CD36, a membrane protein expressed in inflammatory cells, binds to oxidized low-density lipoprotein (oxLDL), thereby activating the inflammatory response and accelerating the development of atherosclerosis [54,55]. In another study in hypercholesterolemic mice, PAI was used to longitudinally monitor atherosclerotic lesions to assess plaque lipids, collagen, macrophage content, and endothelial permeability and correlated with the histological analyses, validating the potential of the protocol for preclinical testing of therapeutic interventions [44].

PAI also has its limitations. Optical attenuation restricts imaging depth to a few centimeters within biological tissues [35,43]. Furthermore, ultrasound waves encounter substantial attenuation and phase distortion when traversing thick bones, compromising image quality and accuracy [30,35,43]. Nevertheless, PAI is a promising tool for the non-invasive and intravascular assessment of atherosclerotic plaque and calcification.

## 6. Light-Sheet Fluorescence Microscopy (LSFM)

LSFM offers a high-resolution, ex vivo approach for studying cardiovascular calcification and plaque. While its in vivo application is limited by tissue penetration [25,28,56,57,58,59,60,61,62,63], LSFM-based technology has been used to study tumors in vivo in animal models [88]. It provides rapid high-resolution imaging of large biological specimens with minimal photodamage [58,59], making it particularly suitable for long-term studies of live tissues, such as tracking the development and progression of cardiovascular diseases in zebrafish [60,89,90,91]. Furthermore, the capacity of LSFM for multiscale imaging allows researchers to examine both the macroscopic architecture and microscopic cellular details of cardiovascular structures [56,58,59]. Its fundamental principle involves illuminating a specimen with a thin sheet of light, allowing selective excitation of a single plane within the sample, while a perpendicular detection system captures the emitted fluorescence [48,56,58]. This setup significantly reduces the background noise compared to traditional widefield microscopy and enhances the axial resolution [48,56]. LSFM operates by scanning a light sheet through the sample while collecting fluorescence emissions orthogonally, facilitating high-speed imaging with reduced photodamage [56,58,59]. This allows the detailed visualization of dynamic processes in live specimens, such as zebrafish embryos, which is valuable for studying developmental and physiological phenomena in cardiovascular systems [56,59,60,89,90].

**Figure 5:**
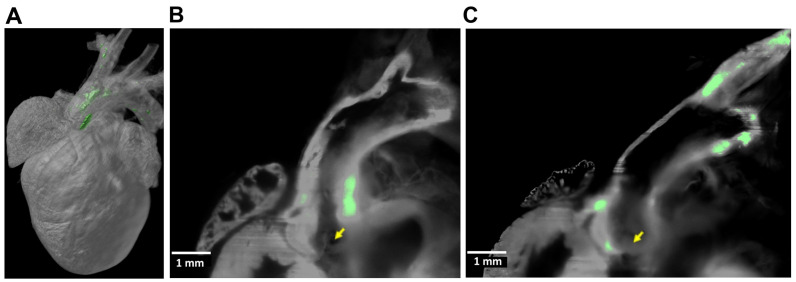
Light-sheet fluorescence microscopy imaging of aortic calcification in mice. (**A**) Three-dimensional reconstruction of calcium deposits located in the aortic root and arch, with a vertical dimension of approximately 1 cm. (**B**,**C**) Two-dimensional raw data, highlighting the distribution of calcium minerals within the aortic root (**B**) as well as in the aortic valve, the lesser curvature of the arch, and the innominate artery (**C**). Yellow arrows in these panels indicate the aortic valve cusps. The green pseudocolor represents values that are 76% of the background tissue autofluorescence. Adapted with permission from Hsu et al. (Ref. [28]).

Ex vivo light-sheet microscopy has been applied in the study of cardiovascular calcification and atherosclerotic plaque [25,28,57,61,62,63]. The imaging of large biological specimens, such as whole organs and tissues, requires optical clearing to allow the light to penetrate the sample more effectively, for clear, detailed images without the need for physical sectioning [25,28,49,57,61,62,63,90,91]. Its ability to image large volumes quickly and with high resolution is particularly advantageous for capturing intricate details of cardiovascular structures. Our team has used light-sheet imaging to demonstrate the spatial distribution of atherosclerotic calcification in the aortas of aged hyperlipidemic mice (Figure 5) [28]. Other studies have employed tissue clearing and light-sheet microscopy to achieve high-resolution, 3D reconstructions of atherosclerotic plaque and neointimal growth in mouse models, revealing detailed plaque morphology and perivascular angiogenesis [51,61,62]. This novel approach allows a comprehensive volumetric analysis, identifying irregularities in plaque volume, geometry, surface structure, and spatial positioning within arteries, providing significant insights into the mechanisms underlying vascular remodeling and the progression of atherosclerosis. Recently, the integration of LSFM with deep learning has been demonstrated to be effective for the detailed 3D characterization and quantification of atherosclerotic plaque in mouse aortas [25]. Their methodology allowed nondestructive imaging and automated segmentation, revealing the highest plaque accumulation in the aortic arch and brachiocephalic artery and insights into plaque composition and immune cell infiltration. Similarly, an approach combining LSFM with machine learning and virtual reality was developed to enhance the analysis of arterial lesions [63]. This method enables the detailed 3D visualization and automated segmentation of atherosclerotic plaque and neointimal hyperplasia, significantly reducing manual processing time and improving the precision of disease quantification in cardiovascular research.

In summary, LSFM offers a high-resolution approach for studying cardiovascular calcification and plaque ex vivo. The future development of LSFM is likely to focus on expanding its utility beyond the current ex vivo paradigm and refining its imaging capabilities to approach true in vivo applications. One key direction will be the engineering of novel illumination and detection strategies to improve light penetration into deeper tissue layers. For example, utilizing near-infrared excitation, advanced tissue clearing, and optimized optical designs will enable LSFM to visualize and quantify blood flow dynamics in the heart’s microcirculation with unprecedented clarity, providing valuable insights into how calcification and plaques affect hemodynamics.

## 7. Multimodal Approaches

Multimodal approaches combining the imaging techniques described above allow for a more comprehensive assessment of plaque composition, inflammation, and calcification, providing a more holistic understanding of atherosclerotic disease development [64,71].

Integrating CT with PET imaging provides a comprehensive methodology for studying atherosclerotic plaque [28,50,53,64,68]. Our group previously used µCT with ^18^F-NaF µPET to investigate the effects of exercise and teriparatide on aortic calcification in the hyperlipidemic mouse models, revealing insights into the potential impacts on plaque stability (Figure 1 and Figure 2) [28,64]. While µCT offers the ability to detect calcification with high resolution, the combined use of ^18^F-NaF µPET imaging, in which the ^18^F-NaF is taken up at the exposed surface areas of metabolically active sites of calcification, allows for the assessment of calcification morphology. Fused µPET/µCT imaging was performed before and after the interventions, to follow the effects of these interventions on the calcification extent and morphology, as discussed in further detail in the PET section [28,64]. Elevated perivascular-adipose-tissue-related imaging markers correlate with early atherosclerotic changes. PET/CT has been utilized to highlight the role of PVAT in capturing vascular inflammation and remodeling processes in *Apoe*^−/−^ rats. Other studies using targeted approaches also enable the detailed visualization of atherosclerotic plaque at different developmental stages for a comprehensive view of plaque progression in mice. In one study, LSFM was combined with PET/CT imaging to investigate the localization of translocator protein (TSPO) in atherosclerotic lesions for the progression of atherosclerosis [57]. In another study in *Apoe*^−/−^ mice, in vivo PET/CT imaging revealed the focal and specific uptake of the 89Zr-labeled probe in atherosclerotic plaque, as confirmed by ex vivo autoradiography and immunohistochemical analyses [53]. Additionally, zirconium-89 (^89^Zr-DFO-Gal3-F[ab’]2 mAb PET/CT imaging has been used to identify high-risk plaque [53].

The integration of PET with MRI has enhanced the ability to correlate molecular imaging findings with anatomical and functional data [28,40,52,64,71], since PET provides functional imaging, while MRI offers high-resolution anatomical imaging. Their integration improves diagnostic performance, allowing for a more detailed assessment of atherosclerotic plaques, including plaque composition, size, and vulnerability [40,46,74,75,92]. Studies have explored the viability of simultaneous PET/MRI for detecting regions of inflammation within vulnerable atherosclerotic plaque in mice, using tracers such as radiolabeled maleylated human serum albumin to identify macrophage-rich lesions [26,52,93]. Beyond inflammatory plaque detection, additional studies have demonstrated a correlation between PET/MRI imaging findings and histological and molecular analyses [40,92]. By using PET to visualize metabolic activity and MRI to assess the structural integrity of the arterial wall, researchers are able to cross-validate imaging findings with ex vivo tissue analysis [40,92]. One study has examined the use of DCE-MRI alongside FDG-PET/CT to assess the effects of pioglitazone on vascular inflammation in atherosclerotic rabbits and demonstrated its potential for evaluating the effects of anti-inflammatory treatment in vivo using this modality [78].

The multimodal capability of PAI, especially when combined with other imaging modalities such as IVUS, enhances its diagnostic power by providing both structural and compositional information [84,85,86,87]. Intravascular PAI (IVPA) combines PAI with intravascular ultrasound (IVUS), providing both anatomical information and functional imaging of the artery wall [30,84,85,86]. IVPA can accurately identify and characterize the composition of atherosclerotic plaque, including lipid cores, fibrous caps, and calcification morphology [30,43,84,85,86,87]. The combination of IVPA and IVUS enhances the assessment of plaque stability and guides therapeutic interventions [30,43]. Innovative advancements have been made in the development of high-sensitivity IVPA catheters, which have been successfully applied for the real-time imaging of lipid distribution within rabbit aortas under clinically relevant conditions at imaging speeds up to 16 frames per second and have shown promising results for accurate lipid localization and quantification [84]. In one study, IVPA was used in miniature swine to visualize lipid-rich plaque in vivo [85]; IVPA and IVUS were combined to enhance the structural and compositional imaging of plaque, highlighting its potential for clinical translation. In addition, the combination of dual-modality IVPA and four-dimensional ultrasound imaging in murine atherosclerosis established a correlation between altered hemodynamics and lipid deposition [86]. That study underscored the ability of IVPA to provide detailed morphological and compositional insights into plaque development. Furthermore, a recent study highlighted advancements in catheter design and imaging techniques that facilitated the comprehensive imaging of the arterial wall [87]. That study presents the translational potential of IVPA technology for clinical use in identifying vulnerable plaque. Altogether, there has been significant progress in the in vivo real-time imaging of lipid-rich plaque using IVPA technology, making it potentially valuable for real-time assessment of plaque stability and guiding therapeutic interventions, such as directing coronary stent implantation.

## 8. Implications and Future Perspectives

Advanced imaging techniques are becoming increasingly valuable in the more precise assessment of vulnerable plaque by detecting early-stage calcifications that are not always visible using traditional imaging methods. Molecular imaging modalities, particularly when coupled with high-resolution techniques, may aid in identifying high-risk lesions and subsequently allow for the in vivo evaluation of therapies that target these plaque characteristics. Interlinking imaging results with discrete molecular pathways of calcification can eventually lead to the development of innovative anti-calcification therapies targeting root mechanisms rather than addressing late-stage disease. For example, the use of nanoparticles can not only enhance imaging resolution but also provide a means to deliver therapeutic agents to visualize and treat disease in a highly targeted fashion. Multimodal imaging allows researchers to gain a more comprehensive view of the atherosclerosis and calcification, leading to better risk assessment and therapeutic strategies. Future research may prioritize the development of more accessible and cost-effective imaging methods as well as the validation of novel imaging techniques in clinical settings. Additionally, the integration of imaging with omics approaches such as spatial transcriptomics may provide deeper insights into the molecular drivers of calcification.

Artificial intelligence and machine learning are transforming the analysis of advanced imaging modalities by enhancing diagnostic precision and clinical utility. These technologies excel in processing complex multimodal imaging data, automating tasks like plaque segmentation and calcification detection, and integrating structural, functional, and molecular information with phenotypic data for comprehensive risk assessment. For instance, with advanced data processing, artificial intelligence, and machine learning algorithms, IVPA datasets can be analyzed rapidly to provide automated plaque characterization and risk stratification.

Alternative methods are also advancing the field of atherosclerosis imaging. For example, ex vivo human tissue incubation techniques, such as those applied to carotid plaques, provide critical insights into plaque biology. Studies utilizing ^18^F-NaF µPET and high-resolution imaging have demonstrated the capacity to localize microcalcifications in plaques, showing heterogeneity in calcification processes not detected by standard CT scans. Similarly, incubation of carotid endarterectomy specimens with novel radiotracers like ^18^F-RGD-K5 has revealed detailed angiogenic activity within the plaques, correlating molecular findings with imaging and histopathological analysis. These methods help to bridge the gap between preclinical animal studies and clinical imaging, offering a closer representation of human pathology.

Nonetheless, the translation of the imaging of animal models to the clinical setting remains a challenge. Financial, regulatory, and equipment-related limitations may pose restrictions on the use of these techniques in clinical research and practice. Furthermore, the physiological and anatomical differences between animal models and humans can impact the translational potential of these findings. For instance, microcalcifications and vascular remodeling detected in smaller-scale animal models may not accurately reflect the complexity of human cardiovascular conditions, which often involve multifactorial disease processes. Safety considerations also play a critical role in the adoption of these advanced imaging modalities. Techniques such as PET imaging pose concerns related to radiation exposure and potential toxicity. The approval process for new radiotracers and imaging agents is often lengthy and resource-intensive, requiring extensive validation in both preclinical and clinical studies to ensure safety and efficacy. Future efforts may be focused on the continued development of imaging technologies that deliver not only higher resolution and specificity but also the advancement of techniques that could be feasible in clinical medicine.

## Figures and Tables

**Table 1:** Comparison of different imaging techniques for vascular plaque in animal models.

Imaging Technique	Species	Advantages	Disadvantages
µCT	Mouse [15,16,17,18,19,20,21,22,23,24,25,26] Rat [27] Rabbit [23,28,29,30] Pig [31,32]	High spatial resolution Microcalcification detectable Contrast-agent-free	Poor soft-tissue contrast High radiation dose Motion artifacts
PET	Mouse [18,19,20,21,22,23,24,25,26,33,34,35,36] Rat [36] Rabbit [23,30,37,38,39] Pig [40] Baboon [41]	High sensitivity Inflammatory regions of atherosclerotic plaque detectable Functional imaging Dynamic imaging	Low spatial resolution Radiation risk Long scanning time Dependence on radiolabeled tracers
MRI	Mouse [34,35,36,42,43,44] Rat [36,45] Rabbit [30,39,46,47] Pig [40]	Excellent soft-tissue contrast No ionizing radiation Detailed plaque characterization	Long scanning time Motion artifacts
Photoacoustic Imaging (PAI)	Mouse [48,49,50] Rabbit [51,52] Pig [53]	High contrast and specificity Deep tissue penetration No ionizing radiation Real-time imaging	Attenuation and distortion in bone Limited imaging depth Technical complexity
Light-Sheet Fluorescence Microscopy (LSFM)	Zebrafish [54,55,56,57] Mouse [20,25,56,58,59,60,61,62,63]	High spatial and temporal resolution Minimal photodamage Fast imaging speed	Limited deep-tissue imaging Complex sample preparation

## Data Availability

No new data were created or analyzed in this study.
